# Case Report: Robot-assisted gait training with the wearable cyborg hybrid assistive limb 2S size in three children with cerebral palsy

**DOI:** 10.3389/fresc.2025.1545105

**Published:** 2025-03-24

**Authors:** Kazushi Takahashi, Hirotaka Mutsuzaki, Tomohiro Nakayama, Mayumi Matsuda Kuroda, Kazunori Koseki, Kenichi Yoshikawa, Junko Nakayama, Haruka Oguro, Ryoko Takeuchi, Masafumi Mizukami, Hiroki Watanabe, Aiki Marushima

**Affiliations:** ^1^ Department of Physical Therapy, Ibaraki Prefectural University of Health Sciences Hospital, Ami, Japan; ^2^ Center for Medical Science, Ibaraki Prefectural University of Health Sciences, Ami, Japan; ^3^ Department of Orthopedic Surgery, Ibaraki Prefectural University of Health Sciences Hospital, Ami, Japan; ^4^ Department of Pediatric, Ibaraki Prefectural University of Health Sciences Hospital, Ami, Japan; ^5^ Department of Physical Therapy, Ibaraki Prefectural University of Health Sciences, Ami, Japan; ^6^ Department of Physical Therapy, Faculty of Rehabilitation, R Professional University of Rehabilitation, Tsuchiura, Japan; ^7^ Department of Neurosurgery, Institute of Medicine, University of Tsukuba, Tsukuba, Japan; ^8^ Cener for Cybernics Research, Institute of Medicine, University of Tsukuba, Tsukuba, Japan

**Keywords:** robot-assisted gait training, hybrid assistive limb, cerebral palsy, three-dimensional gait analysis, electromyography

## Abstract

**Introduction:**

Recently, robot-assisted gait training (RAGT) has attracted attention as a rehabilitation method to efficiently improve walking function. The purpose of this case report is to examine whether there is a change in gait function after RAGT with HAL in children with cerebral palsy (CP).

**Methods:**

Three children with CP participated in this study. Case 1 was an 8-year-old boy with Gross Motor Function Classification System (GMFCS) level II. Case 2 involved a 9-year-old girl with a class IV GMFCS. Case 3 was that of a 10-year-old boy with class IV GMFCS. RAGT was conducted once a day for 20 min for a total of 11–12 sessions. Gait was assessed before and after RAGT. We assessed using three-dimensional motion analysis and surface electromyography (sEMG). The sEMG signals were recorded from the bilateral tensor fascia lata, gluteus maximus, semitendinosus, and rectus femoris.

**Results:**

All cases showed changes in the joint angle and muscle activity in the lower limbs before and after RAGT. In Case 1, the maximum hip extension angle increased from −10.6° to −4.1° at the terminal stance, and the average muscle activity of the gluteus maximus in the right stance phase increased from 22.4% to 30.2%. In Case 2, the maximum extension angle of the left knee joint increased from −43.0° to −26.9°. In Case 3, the maximum hip extension angle increased from −39.9° to −25.9° on the left side and from −35.1° to −18.7° on the right side; the maximum knee extension angle increased from −55.7° to −38.8° on the left side and from −52.1° to −36.9° on the right side.

**Discussion:**

A Case 1 had significant hip flexion during gait, but RAGT with HAL emphasized hip extension motion and enabled an efficient gait. As a result, the maximum hip extension angle increased, and the activity of the gluteus maximus muscle in the stance phase increased. Cases 2 and 3 had greater hip and knee joint flexion angles, however increased knee extension angles after RAGT. The increased hip and knee joint extension angles during the stance phase increased the propulsive force propelling the walker forward.

## 1 Introduction

Gait disturbances due to cerebral palsy (CP) cause abnormal gait patterns typified by equinus, scissors, and crouch gaits, resulting in reduced speed and endurance (1, 2). As children with cerebral palsy improve their gait function by improving the joint range of motion and coordination in the lower limbs, their daily and quality of life also improve (3, 4). Gait disturbance in children with CP is an important issue in pediatric rehabilitation.

Recently, robot-assisted gait training (RAGT) has attracted attention as a rehabilitation method to efficiently improve gait and balance. Impaired gait and balance are primary concerns for individuals suffering from neurological disorders. RAGT has been shown to increase cortical activity in motor-related areas and improve neuroplasticity in patients following brain injury (5, 6). Additionally, active participation RAGT supports motor learning and functional improvement (7). RAGT is a promising neurorehabilitation intervention that improves gait function and has demonstrated success in pediatric rehabilitation (8, 9). We have examined the efficacy and safety of RAGT using a wearable cyborg Hybrid Assistive Limb ® (HAL) (2S size, HAL-FC01, Cyberdyne, Tsukuba Japan) in children with CP or spinal cord disorder, and reported that it improves gross motor function (10). However, while previous studies have examined the effects of RAGT using HAL on gait speed, stride length, and cadence, they have not investigated changes in gait patterns or lower extremity muscle activity. This case report is a secondary analysis of three of the seven study participants, and the purpose of this case report was to verify whether there was any change in gait function after RAGT with HAL. We assessed the efficacy of RAGT with HAL using Three-dimensional motion analysis (3D-MA) and surface electromyography (sEMG).

## 2 Materials and methods

### 2.1 Participants

CP is classified based on the type of movement disorder, areas of disability, and severity. Movement disorders are classified as spastic, athetotic, and ataxic. The spastic type, the most common, is characterized by spasticity. The spastic type is further classified into quadriplegia, diplegia, and hemiplegia, depending on the site of the disorder. The athetotic type involves sustained or intermittent involuntary movements and asymmetric posture. The ataxic type is characterized by tremor and dysmetria, primarily due to extrapyramidal symptoms and cerebellar affection (11). GMFCS is a severity classification of mobility in cerebral palsy, classified into five levels: Level I, walking without limitations; II, walking without walking aids; III, walking with walking aids; IV, limited mobility on one's own; and V, very limited automatic mobility, even with the use of power wheelchairs (12).

**Figure 1:**
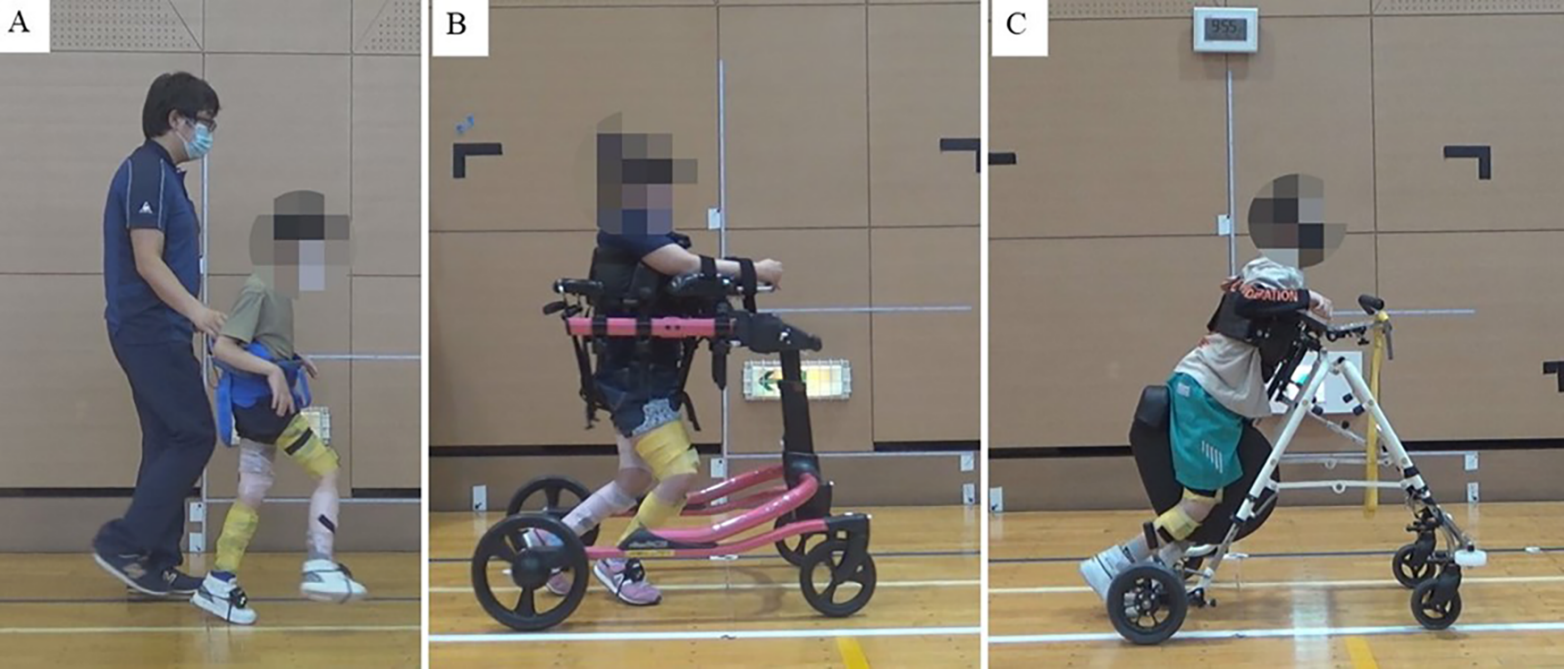
Walking in cases 1–3. **(A)** Case 1: He could walk without a walking aid. However, a fall prevention belt was used for evaluation, **(B)** Case 2: The patient walked using a walker with a seat (Pacer Gait Trainer, Rifton, NY, USA), **(C)** Case 3: The patient walked using a walker with a seat (PROSTAR, Kisaku kobo, Hukono, Japan).

Three children with CP participated in this study. Case 1 was an 8-year-old boy (height, 121 cm; weight, 21.8 kg) with a Gross Motor Function Classification System (GMFCS) level II. The case presented with diplegia and ataxia (Figure 1A). The patient presented CP due to severe birth asphyxia, and had been receiving outpatient physical therapy since he was 7 months old. There was no history of Botox or other spasticity treatments, nor orthopedic surgery. Case 2 was that of a 9-year-old girl (height, 128 cm; weight, 35.6 kg) with class IV GMFCS. Born with extremely low birth weight and diagnosed with periventricular leukomalacia, she was quadriplegia (Figure 1B). She received weekly outpatient physical therapy. At age 8, she underwent a femoral varus derotational osteotmy combined with pelvic osteotomy for left-side hip dislocation. Case 3 was that of a 10-year-old boy (height, 126 cm; weight, 20.8 kg) with class IV GMFCS. He was born prematurely and diagnosed with periventricular leukomalacia and intraventricular hemorrhage. The disability was caused by a combination of quadriplegia and athetosis (Figure 1C). He received weekly outpatient physical therapy and there was no history of Botox or other spasticity treatments, nor orthopedic surgery.

The study protocol was designed according to the Declaration of Helsinki and relevant ethical guidelines for clinical research. This study was approved by the Tsukuba University Clinical Research Review Board (TCRB19-025) and the Ibaraki Prefectural University of Health Sciences (e261). This study was conducted at Ibaraki Prefectural University of Health Sciences Hospital from July 2020 to April 2021.

### 2.2 Robot-assisted gait training (RAGT)

**Figure 2:**
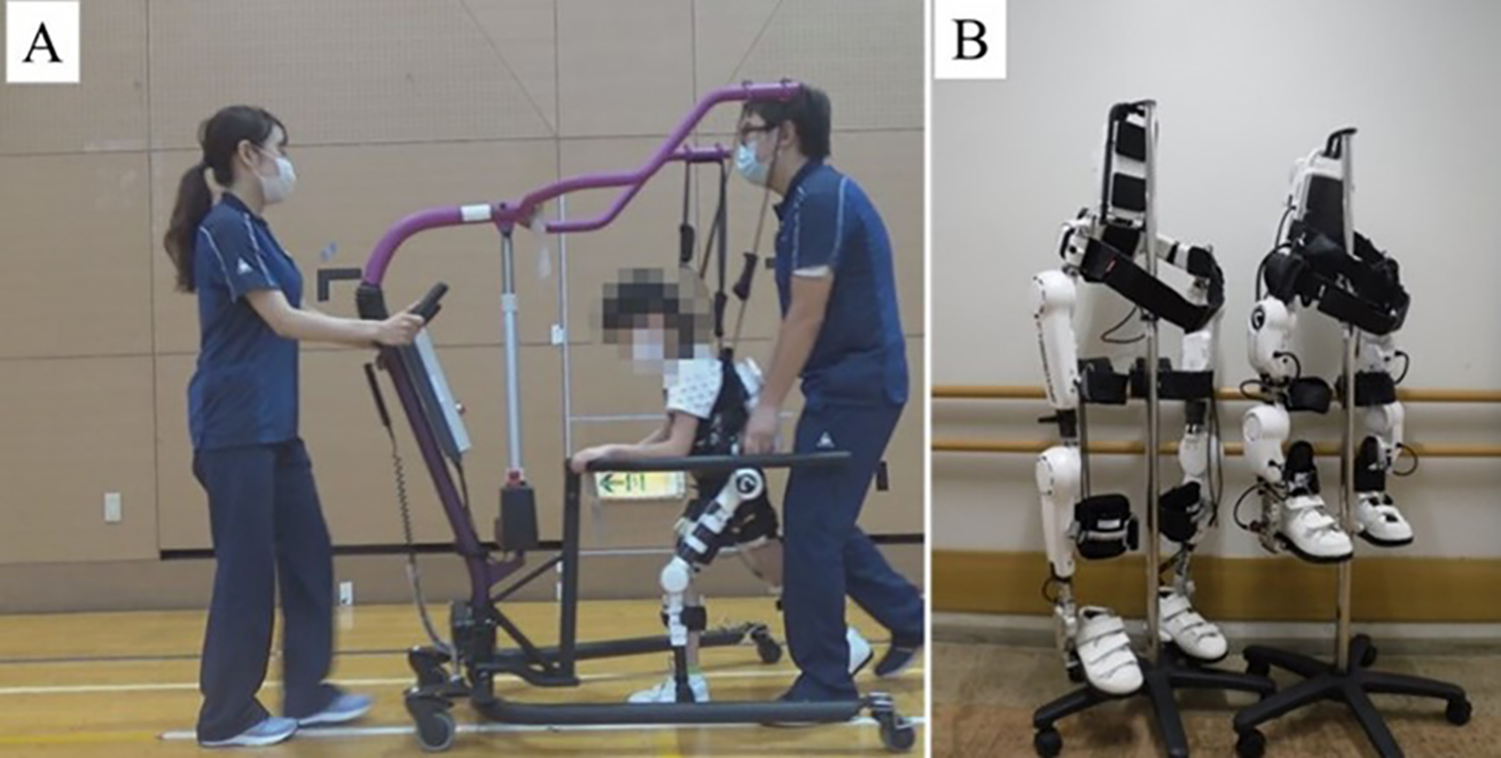
Devices used. **(A)** Scenery of RAGT (HAL 2S size, HAL-FC01, and All-in-One Walking Trainer), **(B)** Size Differences between HAL S and HAL 2S sizes. HAL, Hybrid Assistive Limb ®; RAGT, robot-assisted gait training.

This study used a wearable cyborg HAL for the RAGT (Figure 2A). The HAL is the world's first wearable robotic device. The HAL takes information from the bioelectrical signals generated during the patient's muscle movements and the floor reaction force inside the shoes, and the HAL's power unit assists the wearer's movements based on this information. HAL facilitates muscle activity and movement by providing motion support based on the wearer's voluntary movements (13, 14). HAL can only be used by those 150 cm tall or taller, although a new 2S size (adaptable height of 100–150 cm) has been developed for children (15). This 2S size HAL was used in this study (Figure 2B).

RAGT was conducted once a day for 20 min for 11–12 sessions, over 5–6 weeks. The study protocol allowed participants to choose whether the study would be conducted in an inpatient or outpatient setting. However, all three participants were hospitalized and participated in the study. The hospitalization was solely for participation in the study, with no orthopedic surgery or other spasticity treatments performed. Additionally, participants were prohibited from rehabilitation outside the RAGT for the duration of the intervention (10).

HAL was controlled using the Cybernic Voluntary Control mode in accordance with previous studies (10, 15, 16). The Cybernic Voluntary Control mode controls the assist torque of the HAL based on bioelectrical signal information (13). HAL can set the maximum percentage of torque to be applied to assist and the range of motion angles of the lower limbs to ensure that robotic assistance is safe. Also, the magnitude of the assist torque and the assist balance during flexion and extension can be finely adjusted. The lower limb movements of children with CP during gait are unequal from side to side. Also, gait varies with walking speed and degree of fatigue. The amount of torque and assist balance for each joint were fine-tuned in consultation with the patients. To prevent falls during RAGT, an All-in-One Walking Trainer [Ropox A/S, Naestved, Denmark] or the participant's own posture control walker was used (Figure 2A).

### 2.3 Outcome measures

Gait was assessed before and after RAGT. For gait measurement, participants walked at a self-selected walking speed along a 14-m walking track, and joint angles and muscle activity during gait were calculated from 10 gait cycles. RAGT was not used during gait assessment, and participants used their usual gait method, either independent walking or using a walker for support.

To encourage patient engagement, this study used the Canadian Occupational Performance Measure (COPM) to set goals with participants and their families. COPM is an assessment scale that scores changes in clients' own perceptions of their occupational performance and has been used in many studies (17).

#### 2.3.1 Three-dimensional gait analysis

Gait analysis was performed using myoMotion (Noraxon USA, Scottsdale, AZ, USA), a wireless inertial measurement unit (IMU) system. The myoMotion consists of a receiver and seven IMUs for the lower body (pelvis and bilateral thighs/lower limbs/feet). The IMUs size is 37.6 mm × 52.0 mm × 18.1 mm, and weight is 34 g. Each IMU has a local coordinate system that measures the acceleration in three directions: yaw, pitch, and roll. The body segment to which the IMU was attached was assumed to be a single rigid body and each body segment was considered to be a rigid unit with interconnected joints. The myoMotion sampling rate was 100 Hz and calibration was performed in the sitting position. Heel contact and toe-off judgments during walking were discriminated using the contact detection algorithm of the software myoRESEARCH 3.16.86 (Noraxon USA, Scottsdale, AZ, USA), and the joint angles during gait were calculated using the same software.

#### 2.3.2 Assessment of muscle activity using sEMG

sEMG was performed using an Ultium-EMG (Noraxon USA, Scottsdale, AZ, USA). The sampling rate was 2,000 Hz, and the signal was filtered (Butterworth, band pass, 10–500 Hz). The surface electrodes were blue sensors (P-00-S, METS, Tokyo, JPN) with an inter-electrode distance of 2 cm. sEMG installation was performed according to SENIAM international standards (18). The sEMG signals were recorded from the bilateral tensor fascia lata, gluteus maximus, semitendinosus, and rectus femoris. These muscles were selected because they are the muscles from which HAL bioelectrical signals are acquired. The sEMG and 3D-MA data were analyzed synchronously using myoRESEARCH 3.16.86. EMG data were rectified and calculated as root-mean-square values over a 20 ms window and normalized by peak sEMG during walking.

## 3 Results

**Figure 3:**
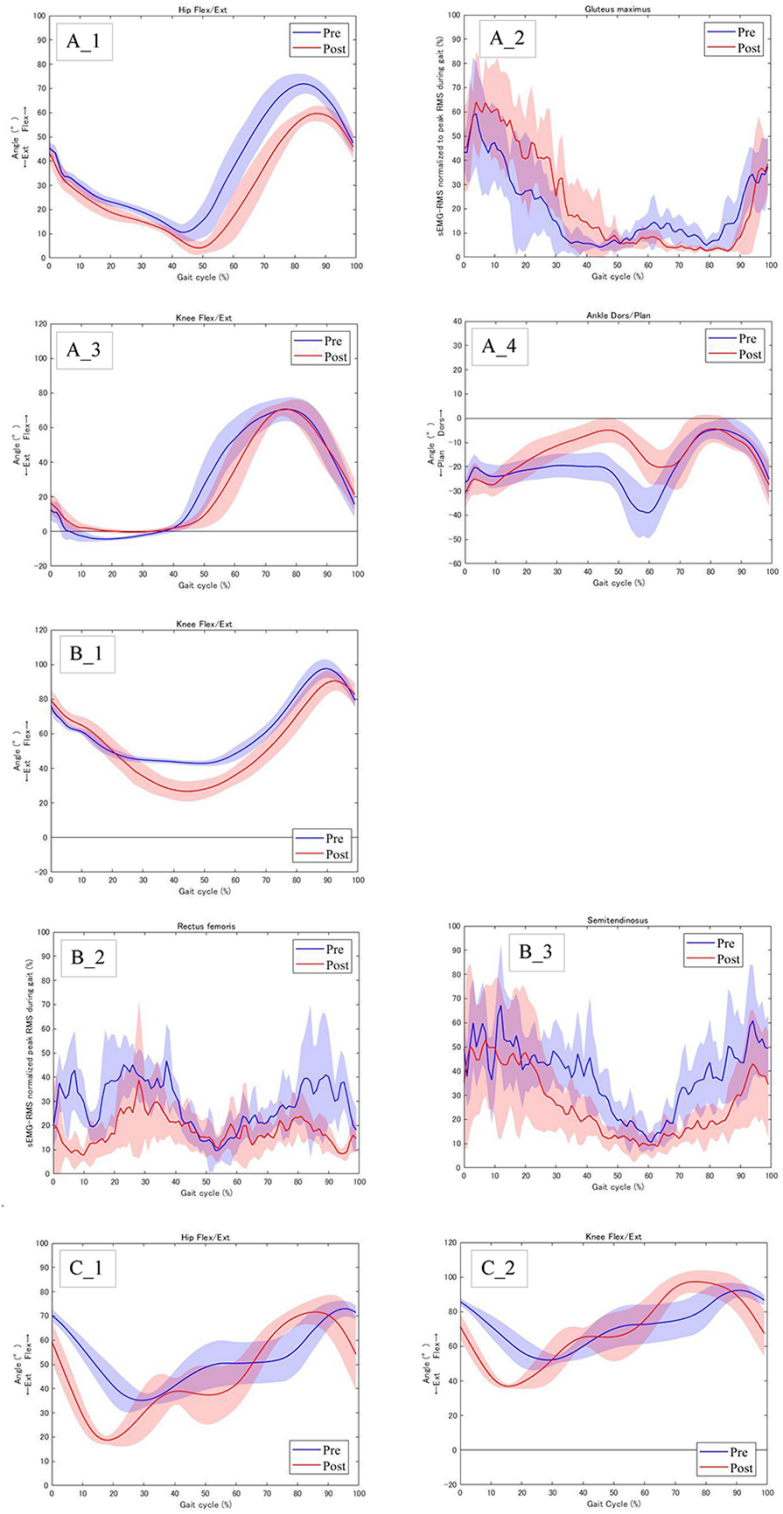
Joint angles and muscle activity of the lower limb before and after RAGT. **(A)_1** Case 1: Right hip flexion/extension angles, **(A)_2** Case 1: Right muscle activity of the gluteus maximus, **(A)_3** Case 1: Right knee flexion/extension angles, **(A)_4** Case_1: Right ankle dorsiflexion/plantar flexion angles, **(B)_1** Case 2: Left knee flexion/extension angles, **(B)_2** Case2: Right muscle activity of the rectus femoris, **(B_3)** Case 2: Right muscle activity of the semitendinosus. **(C)_1** Case 3: Right hip flexion/extension angles, **(C)_2** Case 3: Right knee flexion/extension angle.

All patients showed changes in the joint angle and muscle activity in the lower limbs before and after RAGT. Case 1 had GMFCS level II and could walk alone Figure 1A. In this case, the maximum hip extension angle increased from −10.6° to −4.1° at the terminal stance (Figure 3A_1), and the average muscle activity of the gluteus maximus in the right stance phase increased from 22.4% to 30.2% (Figure 3A_2). The joint angle curves of the knee and ankle joints during walking are shown in (Figure 3A_3,4). Cases 2 and 3 had GMFCS level IV and used a walker (Figures 1B,C). Joint angles change from terminal stance to pre-swing. In Case 2, the maximum extension angle of the left knee joint increased from −43.0° to −26.9° (Figure 3B_1). In addition, the muscle activities of the rectus femoris and semitendinosus muscles are shown in (Figure 3B_2,3). In Case 3, the maximum hip extension angle increased from −39.9° to −25.9° on the left side and from −35.1° to −18.7° on the right side; the maximum knee extension angle increased from −55.7° to −38.8° on the left side and from −52.1° to −36.9° on the right side (Figure 3C_1,2). All joint angles and muscle activity diagrams for Cases 1–3 were included in the Supplementary Appendix.

COPM improved performance and satisfaction in all cases. In case 1, performance improved from 3.4 to 6.2 and satisfaction from 2.8 to 6.6; in Case 2, performance improved from 2.2 to 3.8 and satisfaction from 2.0 to 3.0; and in Case 3, performance improved from 0.2 to 3.2 and satisfaction from 0.2 to 3.2.

## 4 Discussion

The advantage of the RAGT is that the wearer is corrected to a good posture by the exoskeleton of the robot and can repeat rhythmic walking movements with a good posture. A systematic review of the effectiveness of robotic rehabilitation for children with CP reported that RAGT was effective in improving motor function and gait (19). The wearable cyborg HAL used in this RAGT assists the wearer's activities by generating assist torque in power units located at the hip and knee joints from bioelectrical signals generated during muscle activity and floor reaction force signals generated during a weight shift (13, 14). Therefore, the HAL can provide active assistance based on the wearer's motor intentions. We previously reported that RAGT using the HAL improved gross motor function in children with CP and spinal cord disorders (10). However, few pediatric studies have examined how RAGT changes lower limb joint angles and muscle activity during gait. In this case report, we investigated whether gait function changed after RAGT using 3D-MA and sEMG.

Case 1 had a GMFCS level II and could walk alone (Figure 1A). In a normal gait, the hip joint makes two movements: extension during the stance phase and flexion during the swing phase. Hip range of motion during walking at self-selected speeds ranges from 40° of flexion to 10° of extension (20). In contrast, the gait pattern of the CP is a gait pattern with significant hip flexion, as typified by jump-knee gait and crouch gait (21). In this case, the hip range of motion during gait was 71.9° of flexion to −10.6° of extension, with a deviation toward increased flexion (Figure 3A_1). RAGT with HAL improved the maximum hip extension angle during terminal stance from −10.6° to −4.1° (Figure 3A_1). To adjust the assist torque of the motor, the HAL can be equipped with a torque tuner to adjust the amount of torque, and a balance tuner to adjust the balance of the assist torque during flexion and extension. The HAL balance tuner was augmented in the extension direction, and the RAGT emphasized hip extension movements to help the patient achieve an efficient gait. Consequently, the hip extension angle in terminal stance increased (Figure 3A_1). Additionally, gluteus maximus activity was increased during the stance phase (Figure 3A_1, 2). The gluteus maximus is the primary hip extensor during the loading response and assists in stabilizing the hip as the body moves forward (22). In Case 1, this increased muscle activity contributed to hip stabilization during anterior body movement and contributed to the greater hip extension angle at terminal stance. The angular curve of the knee joint during gait in Case 1 was similar to the normal independent gait, but the ankle was plantar flexed and equinus during gait (Figure 3A_3, 4). RAGT with HAL resulted in decreased ankle plantar flexion from terminal stance to pre-swing. The HAL assist may have increased hip extension in the terminus stance, increased backward stride length by increasing the center of gravity movement distance, and increased ankle dorsiflexion.

Cases 2 and 3 used a walker with a seat due to severe disability with GMFCS IV, limiting their ability to walk independently (Figures 1B,C). Comparing gait with a walker to normal independent gait is difficult because the motor patterns of the lower limbs are significantly different. The use of an anterior walker in children with CP results in increased hip and knee joint flexion (23). RAGT increased the hip and knee joint extension angles from terminal stance to pre-swing, which increased the propulsive force required to propel the walker forward (Figures 3B_1,C_1,2). Also, CP children have a motor strategy of excessive co-contraction of the agonist and antagonist muscles during standing and walking (24). This prevents smooth movement of the joints and reduces the joint angle range during gait. In cases 2, the rectus femoris and semitendinosus muscles, which agonist and antagonize the flexion and extension of the knee joint, remained contracted and the joint angle range of the knee joint during gait was small. RAGT with HAL reduced muscle activity in the rectus femoris and semitendinosus muscles during gait and increased the angular range of the knee joint (Figure 3B_2,3).

In this case report, we examined whether RAGT with HAL could change gait patterns and lower limb muscle activity in three children with CP and found that it did. Most RAGT studies of children with CP have focused on mild to moderate severity, such as GMFCS II and III (9). In this context, the study included two severely disorder children, but their gait patterns improved. The results suggest that RAGT has the potential to alter gait function in severely disordered children. However, the disabilities of children with CP are diverse and vary in severity. In the future, we would like to increase the number of cases and identify appropriate programs according to the severity of CP and settings for HAL. Additionally, further research is needed to explore whether RAGT using HAL contributes to brain plasticity. The gait biomechanics is an important indicator of the degree of recovery in terms of brain plasticity (25). While previous studies have shown that RAGT using HAL enhances brain plasticity (26), there is a lack of research on pediatric conditions such as CP. Using functional near-infrared spectroscopy and functional MRI could help explore the underlying mechanisms of brain plasticity in RAGT using HAL.

RAGT has been reported as more cost-effective than conventional physical therapy in stroke patients, improving both motor function and gait (27). While no cost-effectiveness studies for children with CP have been published, RAGT has been reported to improve gait and gross motor functions in these children more effectively than conventional physical therapy (9). In Japan, most physical therapy is covered by public insurance, through RAGT using HAL is currently only covered or certain diseases (28, 29). If RAGT using HAL for children with CP were to be covered by public insurance, a comparison of its cost-effectiveness with conventional physical therapy should be conducted.

One limitation of this study was the normalization of the sEMG amplitude. The sEMG amplitude represents the number of action potentials recruited and the firing frequency. Amplitudes are often normalized using standard values because they are strongly influenced by the electrode mounting position, the distance between electrodes, and subcutaneous fat thickness (30). The most common method involves normalizing the peak sEMG obtained during maximal voluntary isometric contraction (MVIC). In this study, we normalized the peak sEMG during walking, but not the MVIC. Children with pediatric disorders of neurological origin, such as CP, have difficulty performing MVIC because they have difficulty voluntarily performing maximal muscle activity (30). Therefore, normalization with the peak sEMG during specific tasks is recommended (30). However, the normalization method using peak sEMG during walking may be influenced by factors other than walking, and caution should be exercised when interpreting the results.

## Data Availability

The original contributions presented in the study are included in the article/Supplementary Material, further inquiries can be directed to the corresponding author.
